# Epidemiology, Clinical Characteristics, and Risk Factors for Running-Related Injuries among South African Trail Runners

**DOI:** 10.3390/ijerph182312620

**Published:** 2021-11-30

**Authors:** Carel T. Viljoen, Dina C. Janse van Rensburg, Evert Verhagen, Willem van Mechelen, Elzette Korkie, Tanita Botha

**Affiliations:** 1 Department of Physiotherapy, Faculty of Health Sciences, University of Pretoria, Pretoria 0028, South Africa; Elzette.Korkie@up.ac.za; 2 Amsterdam Collaboration for Health and Safety in Sports, Department of Public and Occupational Health, Amsterdam Movement Sciences, Amsterdam University Medical Centers, VU University Medical Center, 1081 HV Amsterdam, The Netherlands; e.verhagen@amsterdamumc.nl (E.V.); w.vanmechelen@amsterdamumc.nl (W.v.M.); 3 Sport, Exercise Medicine and Lifestyle Institute (SEMLI), Pretoria 0186, South Africa; christa.jansevanrensburg@up.ac.za; 4 Section Sports Medicine, Faculty of Health Sciences, University of Pretoria, Pretoria 0028, South Africa; 5 School of Human Movement and Nutrition Sciences, Faculty of Health and Behavioural Sciences, University of Queensland, Brisbane, QLD 4072, Australia; 6 Division of Exercise Science and Sports Medicine (ESSM), Department of Human Biology, Faculty of Health Sciences, University of Cape Town, Cape Town 7700, South Africa; 7 School of Public Health, Physiotherapy and Population Sciences, University College Dublin, D04 V1W8 Dublin, Ireland; 8 Department of Statistics, Faculty of Natural and Agricultural Sciences, University of Pretoria, Pretoria 0028, South Africa; Tanita.Botha@up.ac.za

**Keywords:** off-road running, lower limb injury, history of RRI, chronic disease

## Abstract

Trail running involves running on varying natural terrains, often including large elevation gains/losses. Trail running has a high risk of injury, and runners often participate in remote regions where medical support is challenging. The aim of this study was to determine the epidemiology, clinical characteristic, and associated injury risk factors among trail runners. A modified Oslo Sports Trauma Research Center Questionnaire for Health Problems (OSTRC-H) was used biweekly to collect running-related injury (RRI) and training history data prospectively, among 152 participants (males *n* = 120, females *n* = 32) over 30 weeks. We report an overall injury rate of 19.6 RRIs per 1000 h and an RRI mean prevalence of 12.3%. The leading anatomical site of RRIs was the lower limb (82.9%), affecting the knee (29.8%), shin/lower leg (18.0%), and the foot/toes (13.7%). A history of previous RRI in the past 12 months (*p* = 0.0032) and having a chronic disease (*p* = 0.0188) are independent risk factors for RRIs among trail runners. Two in three trail runners sustain an RRI mainly affecting the knee, shin/lower leg, and foot/toes. A history of previous RRI in the past 12 months and a having chronic disease is independently associated with RRI among trail runners. These results could be used to develop future RRI prevention strategies, combined with clinical knowledge and experience.

## 1. Introduction

Trail running is an outdoor activity and one of the most popular running modes under the broader category of off-road running [1]. Trail running is defined as running in natural environments (mountains, forests, deserts, countryside, etc.) on natural variable terrain that involves a maximum of 20% paved roads and has significant elevation gains and losses [1].

Despite the positive health benefits of running and outdoor activities, trail running presents a high risk of injury during race [2,3] and training participation [4]. The majority of trail running literature investigating injury is focused on outcomes related to race participation, with limited studies reporting on training-related injuries [5]. A prospective cohort study among Dutch trail runners reported an overall incidence of 10.7 running-related injuries (RRIs) per 1000 h, showing a higher incidence of overuse (8.1 per 1000 h) vs. acute (2.7 per 1000 h) RRIs [4]. However, acute injuries, such as ankle sprains, [6] contusions, concussion, [7] and tibiofibular joint and meniscus injury [8], are also reported among both male and female trail runners.

Trail runners are often exposed to extreme weather conditions and environmental hazards when running in remote regions, with limited access to medical services [9]. This highlights the need to establish injury risk management strategies among trail runners participating in races and training [9]. Major traumatic injuries receiving delayed medical care in these environments can lead to life-threatening complications and fatalities [10]. There is a lack of literature regarding injury risk factors among trail runners [8], with no prospective cohort studies. Common RRI risk factors identified in the road running literature include age [11,12], sex [11], body mass index (BMI) [12], running experience [8], injury history [11,13], running frequency [11], and chronic disease [14]. A difference in physiological and mechanical stress might be present in trail running vs. road running because of the various off-road-running terrains and larger elevation changes often involved in trail running [15]. It is necessary to investigate if the identified road running risk factors are associated with injury risk among trail runners in order to guide future trail running-specific injury prevention strategies. We also need to investigate whether cross-sectionally determined injury risk factors in trail running, such as chronic disease [16], are still significant when investigated prospectively using longer follow-up periods. Finally, factors specifically related to trail running, such as elevation gains/losses, training surface, and trail running experience, have a potential associated risk that needs further investigation.

The aim of the study was to prospectively determine the incidence, prevalence, and clinical characteristics of RRIs among male and female South African trail runners. We also aimed to investigate whether previously described RRI risk factors also apply to trail running, and if new factors specifically related to trail running are associated with RRI risk.

## 2. Materials and Methods

### 2.1. Study Design

We conducted a prospective cohort study in South Africa from 20 November 2018 to 19 August 2019.

### 2.2. Participants and Data Collection

This study used a dynamic sample, allowing participants to enter the study at different time points. All participants were followed for 30 weeks. We used a convenience sampling method and recruited participants through trail running social media platforms, the South African Trail Running organization, and TRAIL magazine. The inclusion criteria stipulated that participants had to be 18 years or older, have the ability to read and understand English, have access to email, and be training towards a trail race of 21 km or more. Participants were not excluded based on the type or amount of training they were exposed to in preparation for their chosen race. Participants were excluded if they did not complete the baseline and at least one follow-up questionnaire. The study was conducted in accordance with the Declaration of Helsinki and was approved by the Research Ethics Committee of the Faculty of Health Sciences at the University of Pretoria (REC no: 469/2018).

Our study sample included a total of 152 participants, consisting of 78.9% males (*n* = 120) and 21.1% females (*n* = 32). Even though 41.4% (*n* = 63) of runners had more than 5 years of total running experience, only 16.4% (*n* = 25) had similar years of experience in trail running. At the study baseline, a total of 26 (17.1%) participants reported having a chronic disease including hypercholesteremia (28.9%), hypertension (21.1%), asthma (18.4%), hypothyroidism (7.9%), and diabetes (5.3%). Most participants (*n* = 109, 71.7%) sustained an RRI in the 12 months before entering the study, while 53.8% (*n* = 71) had a current RRI upon entering the study.

An analysis was done to determine whether the baseline data significantly differed between males and females (Table 1).

Eligible responders received a link via email, which guided them to an online consent form and baseline questionnaire (online Supplementary File S1). At baseline, we recorded their demographic profile, running experience, training history, current medical conditions, medicine use, current RRI, and history of previous RRI (past 12 months). Subsequently, participants received a link via email every second week to an online follow-up questionnaire on the Qualtrics platform (online Supplementary File S2). These questionnaires recorded self-reported data on (1) the participants’ biweekly training history (total running distance (km), amount of running sessions on trails (n) and road (n), total vertical gain/loss (m), average running pace (min/km), average altitude trained at (m), and amount of hours spent on cross-training (h)); (2) RRI history (anatomical region, RRI type, gradual/sudden onset, mechanism of RRI, and to what extent the RRI affected their running ability), which was reported using a modified Oslo Sports Trauma Research Centre Questionnaire on Health Problems (OSTRC-H) questionnaire; [17] and (3) RRI severity (OSTRC severity score 0–100). Participants not responding to the email within three days received a reminder email with a specific date range, stating the two-week time period the questionnaire referred to.

### 2.3. Health Problem Registration and Classification

A modified version of OSTRC-H [17] was used to prospectively register health problems during the biweekly follow-up periods. As OSTRC-H allows for the recording of sudden onset injury and gradual onset injury, and provides additional information on the location, symptoms, and type of problem [17], it served the purpose of our study. OSTRC-H has a high internal consistency (Cronbach’s alpha = 0.91) [17]. We defined a health problem as an RRI or illness that resulted in modifying the participant’s training. If a respondent reported a health problem, a follow-up question was asked that required them to specify whether their training had to be modified due to “injury” or “illness”. For this study, we only reported injury-related data. Multiple factors can affect performance in trail running and not all symptoms following a trail run can be directly linked to injury. Therefore, we employed training modification as our injury definition. Participants also had to indicate whether their injury had a gradual or sudden onset and whether this was the first time they reported the specific injury through the online injury surveillance platform. A “first-time” injury was recorded as a new injury, while all other injuries (injuries reported previously during the 30 weeks follow-up period) were recorded as “recurrent” injuries. An experienced sports physiotherapist (CTV) evaluated the data of each reported injury to ensure the injury was running-related. If any clarity was needed on the reported injury, the participant was contacted to obtain further information.

### 2.4. Outcome Measures

We reported the biweekly mean prevalence (% of runners; 95% CI) and injury rate (per 1000 h) for new/recurring RRIs and sudden/gradual onset RRIs over 15 two-week periods. The frequency of RRI characteristics (*n*; % of RRIs) was reported in the categories of the anatomical region, body area, tissue type, and pathology type, as stipulated by the 2020 International Olympic Committee (IOC) consensus statement regarding methods of reporting epidemiological data on injury in sports [18]. Running-related injury severity was calculated through the OSTRC-H injury severity score (0–100), allocating a score (0–25) to each response on four key questions regarding an injury [17]. In the modified OSTRC-H questionnaire, four questions related to how RRI affected the participants’ (1) training/race participation, (2) running performance, (3) severity of their health complaint, and (4) pain while running (online Supplementary File S2, question 2–5). Each question added up to 25, contributing to a composite score of 100. A higher OSTRC-H injury severity score indicates a higher severity of the injury [17].

With limited available evidence regarding injury risk factors in trail running, we used common injury risk factors identified in the general running literature, including the participants’ demographic profile (sex, age (years) [11] and BMI [12,13]), biweekly running exposure [11] (number of running sessions, hours of running, and running distance (km)), running experience [8] (years of actively participating in running), and RRI history [11,13] (current RRI and RRI during the past 12 months). Additionally, factors unique to trail running were investigated as injury risk factors: running surface (trail, road, grass, tartan, and treadmill), trail running experience (years of actively participating in trail running), biweekly trail running exposure (number of trail running sessions), and elevation gains/losses (biweekly ascent (m) and descent (m) during running sessions). In certain urban areas of South Africa, access to trails is challenging, and trail runners may use cross-training as an adjunct training modality. Therefore, cross-training (cycling, weight training, swimming, rowing, and functional strength training) were investigated as an injury risk factor in the univariate analysis. In addition, chronic diseases among endurance runners are common and have displayed an associated risk for injury [19]. A history of chronic disease was thus investigated as an injury risk factor in this study.

### 2.5. Statistical Analysis

The statistical program R was used for the analysis of the data [20]. The response rate (%) for each of the two-week periods (total of 15 periods) was calculated by dividing the total number of respondents by the number of invites for each specific period and was then averaged across the 15 time periods. For example, if we had only three time periods and 100 participants were contacted, but only 80 participants responded in the first time-period (80/100 × 100 = 80%), 70 responded in the second time period (70/100 × 100 = 70%), and 85 responded in the third time period (85/100 × 100 = 85%), then we calculated the average over all three time periods, (80 + 70 + 85)/3 = 78.3%. Instances where participants failed to supply a response for a two-week interval, were considered a “no response” and were treated as such. When calculating specific results for each of these two-week periods, the missing participants were not included in the baseline of participants for that time period. In the risk factor analysis (mixed logistic regression model), the random effect accounted for the repeated measures within each participant. Here, the modelling only considered the available data.

For running exposure, the mean duration (hours) of running was calculated using the specific biweekly period’s average running pace and multiplying it by the total biweekly running distance. For example, if a participant ran an average pace of 06:00 min/km and an average distance of 10 km during a time period, then the duration equals 60 min = 1 h of running exposure for that time period. We used the non-parametric Mann−Whitney U test to explore statistically significant differences between training variables of males vs. females (biweekly frequency or running sessions, distance ran, and duration of running), and the Wilcoxon signed-rank test to investigate for significant differences between gradual vs. sudden onset RRI variables (prevalence and injury rate) (tested at a 5% level of significance). Non-parametric tests were used as the Shapiro−Wilk test found that the data were not normally distributed.

The prevalence and injury rate calculations were similar to Hespanhol Junior et al. (2017) [4]. For each period (two weeks), the prevalence was calculated by dividing the number of participants reporting RRIs during that period by the total number of respondents during the specific period. The mean prevalence and the 95% CI were calculated by summing the prevalences across all two-week periods and dividing the total by the number of two-week periods. The injury rates for all new and recurrent RRIs were calculated by dividing the number of RRIs (all, new, and recurrent) by the total running exposure in hours across all the periods. For each of the two-week periods, the four OSTRC questions were used to calculate a score out of 100, obtaining a severity score per participant. The average severity score was then calculated per anatomical region and body area by taking the severity score for all participants per region/area and dividing it by the number of injuries per region/area. The average OSTRC severity score was subsequently calculated per region/area across all periods.

Risk factor analysis for unique RRIs was completed by using Mixed Effect Logistic Regression models. The severity score was used to determine for which entries the participants had to modify their training. We then classified all entries into two categories: injury (modified training) and no injury (no training modification). Mixed-effects regression models were used to consider the repeated measures of the participants’ replies every two weeks, while all other variables were fixed. Significant factors (*p* < 0.05) from the univariate analysis were further explored in a multivariate model. Odds ratios (PR; 95% CIs) were reported, and a significance level of *p* < 0.05 was accepted.

## 3. Results

### 3.1. Response Rate

We observed a mean participant response rate of 67.4% (95% CI: 59.81–74.93) over 15 time periods. The lowest response rate was recorded in period 15 (37.5%) and the highest response in period 1 (100%).

### 3.2. Running Exposure

Considering all forms of running per two-week period, the mean frequency of running sessions was 6.5 (95% CI 6.0–7.0) and the trail running sessions contributed a mean of 2.6 (95% CI 2.3–3.0) to this total (Table 2). On average, participants ran distances of 70.2 km (95% CI 62.8–77.7) and the running duration was calculated at an average of 6.5 (95% CI 5.8–7.1) h (Table 2).

### 3.3. Running-Related Injuries (Prevalence, Injury Rate, Severity, Anatomical Region, Body Area, and Tissue Type/Pathology Type)

A total of 205 RRIs were recorded among the 152 participants. Of the 1536 questionnaire responses over the 30-week study period, 185 (12.0%) questionnaire responses reported one RRI, 7 (0.5%) reported two RRIs, and 2 (0.1%) reported three RRIs. A total of 102 (67.1%) participants sustained at least one injury.

#### 3.3.1. Prevalence of RRIs

The mean prevalence of all RRIs measured every two weeks was 12.3% (95% CI: 10.2–14.4) (Table 3). Males had a higher mean prevalence of RRIs, 10.5% (95%: 8.6–12.4), than females, 1.8% (95% CI: 1.1–2.6), with a mean difference of 8.7% (*p* < 0.0001). The mean prevalence of all RRIs was not significantly higher for sudden onset (6.4%; 95% CI: 4.9–8.0) compared to gradual onset RRIs (5.9%; 95% CI: 4.6–7.1), with a mean difference of 0.5% (*p* = 0.4212).

#### 3.3.2. Injury Rate

The injury rate for new RRIs was 15.3 RRIs per 1000 h of running (Table 3). Males presented with a significantly higher injury rate (12.7 RRIs per 1000 h of running) than females (3.1 RRIs per 1000 h of running), with an injury rate difference of 9.6 RRIs per 1000 h of running (*p* = 0.0298).

#### 3.3.3. Anatomical Region and Body Area

In Table 4, the RRI frequencies (n; %) of all RRIs are presented in categories of the main anatomical region and body area for RRIs. The average OSTRC injury severity score is further presented for the injured anatomical regions and body areas.

The main anatomical region affected by RRIs was the lower limb (82.9%), followed by the hip/groin/pelvis (5.9%) and the lower back/abdomen (4.9%). The top three body regions in the lower limb region involved the knee (29.8%), followed by the shin/lower leg (18.0%) and the foot/toes (13.7%).

The highest OSTRC injury severity score was reported for RRIs to the thoracic spine/chest (62.6), followed by lower back/abdomen (55.5) and lower limb (47.8).

The majority of tissue types involved in RRIs were muscle/tendon (52.7%), of which the main pathology type were tendinopathies (27.8%), followed by muscle injuries (20.5%) and joint sprains (8.8%) (online Supplementary File S3).

### 3.4. Risk Factors Associated with RRIs among Trail Runners

#### 3.4.1. Risk Factors Associated with RRIs (Univariate Analysis)

Risk factors potentially associated with all RRIs were investigated under the following categories: the demographic profile, running experience, training characteristics, RRI history, and medical history (Table 5).

In the training characteristics category, a higher number of any running sessions (OR = 0.9006; *p* = 0.0002) and total running distance (OR = 0.9956; *p* = 0.0156) were associated with significantly lower odds of sustaining an RRI among trail runners.

A significantly higher odds of sustaining an RRI was noted among trail runners with a history of a previous RRI in the past 12 months (OR = 2.1110; *p* = 0.0020) and among those reporting a chronic disease (OR = 1.9680; *p* = 0.0210).

#### 3.4.2. Independent Risk Factors Associated with RRIs (Multiple Regression Analysis)

In Table 6, the independent risk factors associated with RRIs among trail runners are reported.

Independent risk factors associated with RRIs among trail runners included a history of previous RRIs in the past 12 months (OR = 2.0880; *p* = 0.0032) and having a chronic disease (OR = 2.0390; *p* = 0.0188). A higher biweekly number of running sessions was associated with significantly lower odds of sustaining an RRI among trail runners (OR = 0.8889; *p* = 0.0041).

## 4. Discussion

The main goal of our study was to determine the incidence, prevalence, and clinical characteristics of RRIs among South African trail runners. To our knowledge, this is the first study to investigate injury risk factors among trail runners based on data collected in a prospective cohort study.

### 4.1. Injury Rate and Prevalence of RRIs

We reported an injury rate of 19.6 RRIs per 1000 h of running, with a biweekly RRI prevalence of 12.3% among South African trail runners. In a prospective cohort study among Dutch trail runners followed over six months, Hespanhol et al. reported a lower injury rate of 10.7 RRIs per 1000 h of running, with a higher biweekly RRI prevalence (22.4%) [4]. We further reported a statistically significant higher injury rate in males (12.7 RRIs per 1000 h of running) compared with females (3.1 RRIs per 1000 h of running). This is in contrast to a recent systematic review with meta-analysis and meta-regression that reported no difference in injury rates in all running formats for males compared with females [21]. The small number of female participants in our study affected the reliability of our finding. Future trail running studies should specifically investigate sex differences in injury outcomes using larger sample sizes for females. Hespanhol et al. reported a four-fold higher prevalence for overuse (17.7%) vs. acute (4.1%) RRIs [4]. We showed no statistically significant difference in prevalence for sudden onset (6.4%) vs. gradual onset (5.9%) injuries (*p* = 0.4212). We had a higher number of injured runners at baseline (53.8%) compared with Hespanhol et al. (18.0%) [4]. This may explain the differences in injury rate and prevalence, as a history of previous injury increases the risk for further injury in runners [22].

### 4.2. Anatomical Region and Body Area of RRIs

In our study, the lower limb was the most common anatomical region of RRIs among trail runners (82.9%). This is supported by the findings of a systematic review [5] and a prospective cohort study [4] on injury epidemiology among trail runners. The most common body areas involved were the knee (29.8%), shin/lower leg (18.0%), and foot/toes (13.7%). This was in line with findings among Dutch trail runners, where lower leg (20.3%), knee (18.2%), and foot (14.8%) were the most commonly reported RRIs. Even though a recent systematic review [5] showed similar results, Viljoen et al. mainly included race participation studies where data were collected cross-sectionally, complicating the comparison to our current findings. The slight variation between studies on the top three most reported injured body areas could be due to various running environments and slope gradients resulting in different loading patterns, specifically the lower limb [23]. The increased pressure on the lower limbs during running could explain the higher frequency of lower limb injury, not only among trail runners, but also in orienteers, triathletes, and road runners [24]. These findings highlight the importance of developing future RRI prevention strategies focused on managing the risk for lower limb RRIs.

### 4.3. Independent Risk Factors

Similar to previous studies investigating injury risk factors among road runners [25] and elite athletes [26], we showed that a history of previous RRI is an independent risk factor for injury among trail runners. Trail running literature investigating injury risk factors did not specifically investigate injury history as a possible injury risk factor [8,27]. It seems as if some runners are more injury prone and explanations are still lacking. A possible reason may be that kinematic and motor control deficits exist following injury [28], with a subsequent higher risk of re-injury while running on uneven, changing surfaces. This finding emphasizes the need for rehabilitation through tissue loading among injured trail runners in order to obtain optimal physiological, neural, and structural tissue adaptation [29] before a full return to trail running participation.

Chronic diseases such as hypercholesteremia, hypertension, asthma, hypothyroidism, and diabetes were associated with an increased risk for sustaining an RRI in our study. Previous studies have reported an association between chronic disease and gradual onset injury [30]. Certain medications treating chronic diseases have also been associated with an increased risk for injury. These include tendon ruptures following corticosteroid use [31] statin-induced tendinopathies [32], and enthesopathies following use of fluoroquinolones [33]. These findings should be interpreted with caution. It is beyond the scope of this study to determine whether the increased risk for sustaining an RRI noted was due to the presence of physiological stressors of the disease or the medication used to treat the specific disease. Future studies need to explore the casual nature of chronic disease as an injury risk factor before appropriate injury prevention recommendations can be formulated.

A higher biweekly number of running sessions was associated with lower odds for RRIs in our study, but care should be taken in interpreting this finding. We defined injury as training modification. Therefore, it is reasonable to expect that injured runners in our study have less running exposure compared with healthy runners with no training modification, as a result of the injury definition we used.

To understand injury risk, the non-linearity of risk factors should be considered in order to allow for the impromptu interaction between risk factors over time [34]. Known risk factors in trail running are limited, and no studies account for the complexity of RRIs in this population. Therefore, medical professionals should be mindful of the interaction between risk factors, while using them in combination with their clinical knowledge to design injury prevention strategies for trail runners.

### 4.4. Limitations

In South Africa, most trail running races are not affiliated with Athletics South Africa, and runners can participate without membership to any trail running governing body. Without a reference for the population size, we used a convenience sample and could not determine whether our sample was representative of the South African trail running population. This study specifically studied South African trail runners exposed to environments unique to the geography of South Africa, and care should be taken in generalizing our results to the global trail running community. We used self-reported RRI data; thus, the findings of the tissue and pathology types involved in RRIs should be interpreted with caution. We investigated specific injury risk factors such as biweekly elevation changes and running exposure, where the accuracy of self-reported data could have been influenced by recall bias. Gradual onset injuries could have originated from other sporting activities (cycling, weight training, etc.) and may not be purely running-related. We also acknowledge that sudden onset injuries could have been due to underlying repetitive tissue overload with subsequent acute symptoms. To investigate runners with the intent to run on trails, our inclusion criteria required a runner to train towards a trail run race of at least 21 km or more. However, we acknowledge that certain runners may still perform the largest portion of their training on non-trail surfaces. Limited by our sample size, we could only explore a certain amount of injury risk factors. The methodology used in our study did not allow us to account for the complexity of sports-related injuries. Future studies using larger sample sizes could use a time-to-event analysis to explore varying running exposures such as spikes in running distance.

## 5. Conclusions

Approximately two out of three trail runners sustain an RRI mainly affecting the knee, shin/lower leg, and foot/toes. We report an overall injury rate of 19.4 RRIs per 1000 h of running (males: 12.7 RRIs per 1000 h of running; females: 3.1 RRIs per 1000 h of running) with a higher injury rate, and prevalence noted for sudden vs. gradual onset RRIs. A history of previous RRI in the past 12 months and having a chronic disease were independent risk factors for sustaining an RRI among trail runners. Before our findings are implemented into injury prevention strategies, further research is needed to determine if these risk factors are associated with injury in other trail running populations.

## Figures and Tables

**Table 1:** Baseline data (demographic profile, running experience, medical history, and RRI history) of all study participants (*n* = 152).

Characteristic		All Participants (*n* = 152)	Female (*n* = 32)	Male (*n* = 120)	*p*-Value
Age (yrs)		37.1 (9.1)	35.9 (8.8)	37.4 (9.2)	0.4015
mean (SD)					
Height (cm)		177.6 (8.4)	167.8 (5.6)	180.2 (7.0)	<0.0001 *
mean (SD)					
Weight (kg)		76.3 (11.7)	63.4 (7.3)	79.7 (10.2)	<0.0001 *
mean (SD)					
BMI (kg/m^2^)		24.1 (2.8)	22.52 (2.4)	24.6 (2.7)	<0.0001 *
mean (SD)					
Total running experience *n* (%)	0–2 yrs	37 (24.3%)	10 (31.2%)	27 (22.5%)	0.3962
	>2 to 5 yrs	52 (34.2%)	8 (25.0%)	44 (36.7%)	
	>5 yrs	63 (41.4%)	14 (43.8%)	49 (40.8%)	
Trail running experience *n* (%)	0–2 yrs	66 (43.4%)	12 (37.5%)	54 (45.0%)	0.7445
	>2 to 5 yrs	61 (40.1%)	14 (43.8%)	47 (39.2%)	
	>5 yrs	25 (16.4%)	6 (18.8%)	19 (15.8%)	
Chronic disease *n* (%)	Yes	26 (17.1%)	7 (21.9%)	19 (15.8%)	0.5876
	No	126 (82.9%)	25 (78.1%)	101 (84.2%)	
Current RRI (at study entry) *n* (%)	Yes	71 (53.8%)	19 (63.3%)	52 (51.0%)	0.3248
	No	61 (46.2%)	11 (36.7%)	50 (49.0%)	
	Missing	20	18	2	-
Previous RRI (past 12 months) *n* (%)	Yes	109 (71.7%)	24 (75.0%)	85 (70.8%)	0.8071
	No	43 (28.3%)	8 (25.0%)	35 (29.2%)	

SD—standard deviation; BMI—body mass index; RRI—running-related injury. * Statistically significant.

**Table 2:** The participants’ (*n* = 152) mean (95% CI) running exposure (frequency, distance, and duration of running) over 15 two-week periods.

	All Participants (*n* = 152)	Female (*n* = 32)	Male (*n* = 120)	*p*-Value
Frequency (running sessions/two-week period)	6.5 (6.0–7.0)	6.4 (5.3–7.6)	6.5 (5.9–7.1)	0.7439
mean (95% CI)				
Distance (km/two-week period)	70.2 (62.8–77.7)	59.1 (45.7–72.5)	73.2 (64.5–81.9)	0.1751
mean (95% CI)				
Duration (h/two-week period)	6.5 (5.8–7.1)	5.8 (4.7–6.9)	6.7 (5.9–7.4)	0.4190
mean (95% CI)				

*p*-value: male vs. female study participants; CI: confidence interval.

**Table 3:** (1) Total number (n) of questionnaire responses that reported an RRI (gradual and sudden onset, and new and recurring), (2) prevalence (%), and (3) injury rate (RRIs per 1000 h).

RRI		All	Gradual Onset	Sudden Onset	*p*-Value
All RRIs	Number of questionnaire responses that reported an RRI (n)	194	94	100	
	Prevalence Mean (95% CI)	12.3 (10.2–14.4)	5.9 (4.6–7.1)	6.4 (4.9–8.0)	0.4212
	Injury rate Mean RRIs per 1000 h	19.6	-	10.1	
New RRIs	Number of questionnaire responses that reported an RRI (n)	152	67	85	
	Prevalence Mean (95% CI)	9.7 (7.3–12.0)	4.2 (3.0–5.4)	5.5 (3.9–7.0)	0.0917
	Injury rate Mean RRIs per 1000 h (95% CI)	15.3	-	8.6	
Recurrent RRIs	Number of RRIs registered (n)	42	27	15	
	Prevalence Mean (95% CI)	2.6 (1.9–3.4)	1.7 (0.9–2.4)	1.0 (0.6–1.4)	0.0918
	Injury rate Mean RRIs per 1000 h (95% CI)	4.5	-	2.0	

RRI—running related injuries; CI—confidence interval.

**Table 4:** Anatomical region and specific body area of all RRIs (*n* = 205) among 152 trail runners (% all RRIs and OSTRC injury severity score).

Anatomical Region	Body Area	*n*	% Of All RRIs (*n* = 205)	OSTRC Severity Score(Mean 95% CI)
Head, neck & face	All	4	2.0	-
	Head/face	2	1.0	-
	Neck	2	1.0	-
Upper limb	All	2	1.0	-
	Shoulder	1	0.5	-
	Wrist	1	0.5	-
Thoracic spine/chest	All	7	3.4	62.6 (52.6–72.5)
	Thoracic spine	4	2.0	-
	Chest/ribs	3	1.5	-
Lower back/abdomen	All	10	4.9	55.5 (41.6–69.4)
	Lumbar spine	8	3.9	56.0 (38.8–73.3)
	Abdomen	2	1.0	-
Hip/groin/pelvis	All	12	5.9	46.4 (34.8–58.0)
	Pelvis/gluteal	6	2.9	47.5 (30.8–64.2)
	Hip/groin	6	2.9	45.3 (27.7–62.9)
Lower limb	All	170	82.9	47.8 (44.47–51.2)
	Thigh (posterior)	13	6.3	38.6 (26.3–51.0)
	Thigh (anterior)	4	2.0	-
	Knee	61	29.8	50.0 (44.7–55.3)
	Shin/lower leg	37	18.0	44.1 (38.0–50.2)
	Ankle	27	13.2	50.0 (41.6–58.4)
	Foot/toes	28	13.7	50.5 (40.4–60.71)

RRI—running related injuries; CI—confidence interval.

**Table 5:** The odds ratio estimate (%; 95% CI) and p-value for trail runner race entrants with an RRI according to demographic profile, running experience, training characteristics, RRI history, and medical history (univariate analysis).

Characteristic		Odds Ratio Estimate	95% CI	*p*-Value
Demographic profile				
Age (years)		0.9830	0.9627–1.0030	0.1015
Sex (male/female)		1.2320	0.7736–2.0260	0.3898
BMI (kg/m^2^)		0.9712	0.9054–1.0410	0.4057
Running experience (years of running)				
All running		1.0510	0.9719–1.1340	0.2003
Trail running		1.0290	0.9369–1.1260	0.5425
Training characteristics				
Surface mostly ran on	Trails	1.1090	0.7429–1.6830	0.6169
	Road	1.1060	0.8381–1.4950	0.4895
	Grass	0.9314	0.7153–1.2060	0.5889
	Tartan	0.6942	0.3275–1.2720	0.2785
	Treadmill	0.8926	0.6326–1.2260	0.4947
Number (n) of running sessions per two-week time period	Any	0.9006	0.8512–0.9493	0.0002*
	Trail	0.9481	0.8765–1.0190	0.1649
Total running distance (km) per two-week time period		0.9956	0.9919–0.9990	0.0156 *
Total ascent (m) per two-week time period		1.0000	0.9999–1.0000	0.9524
Total descent (m) per two-week time period		1.0000	0.9999–1.0000	0.8270
Type of cross-training	Cycling	1.053	0.9952–1.1090	0.0575
	Weight training	0.9780	0.8971–1.0590	0.5969
	Swimming	1.0770	0.8968–1.2590	0.3767
	Rowing	1.1170	0.7737–1.5000	0.4986
	Functional training	0.9565	0.8352–1.0660	0.4689
	Pilates	0.6901	0.4216–0.9462	0.0650
	Other	0.9208	0.7821–1.0280	0.2295
	None	0.9264	0.5209–1.3150	0.7305
RRI history				
Previous RRI (past 12 months)		2.1110	1.3400–3.4910	0.0020 *
Current RRI		1.4460	1.0010–2.1360	0.0534
Medical history				
Chronic disease		1.9680	1.1390–3.6440	0.0210 *

* Statistically significant.

**Table 6:** Independent risk factors associated with RRIs among trail runners (multiple regression analysis).

Characteristic		Odds Ratio Estimate	95% CI	*p*-Value
Training characteristics				
Number (n) of running sessions per two-week time period	Any	0.8889	0.8203–0.9634	0.0041 *
Total running distance (km) per two-week time period		1.0010	0.9959–1.0060	0.6991
RRI history				
Previous RRI (past 12 months)		2.0880	1.2790–3.4100	0.0032 *
Medical history				
Chronic disease		2.0390	1.1250–3.6960	0.0188 *

* Statistically significant.

## Data Availability

The data presented in this study are available upon request from the corresponding author.

## Supplementary Materials

The following are available online at https://www.mdpi.com/article/10.3390/ijerph182312620/s1, File S1: Online consent form and baseline questionnaire, File S2: Online follow-up questionnaire on the Qualtrics platform, File S3: The frequency of tissue and pathology types of RRIs among trail runners.
