# Supplementary material for: Epidemiology, Clinical Characteristics, and Risk Factors for Running-Related Injuries among South African Trail Runners
Source: Int J Environ Res Public Health. 2021 Nov 30;18(23):12620. doi: 10.3390/ijerph182312620 (PMC8656810; doi:10.3390/ijerph182312620)
Supplement: Supplementary file 1 [file ijerph-18-12620-s001.zip › Online supplementary File S1.pdf]

Consent & Demographics

Start of Block: Consent

Q1
The aim of this study is to collect data that will help guide future injury/illness prevention strategies among trail runners.

Your participation is truly appreciated among the trail running community.

I have read the participant information (participant info). I understand that I may withdraw from this study at any time without further question.
I hereby consent to participate in this study.

○ I agree (1)

End of Block: Consent

Start of Block: Demographic data

Q7 **Initials and Surname**

____

____

____

____

____

Q9 **Email address** (where we can send your questionnaire every second week)

____

____

____

____

Q27 **ID number** (to identify your data on the follow-up questionnaires)

Q14 **Age**

Q16 **Sex**

- Male (1)
- Female (2)

Q18 **What is your current height (cm)?**

Q20 **What is your current weight (kg)?**

Q21 **Are you planning to participate in a trail run of 21 km or more, during the next 6 months?**

○ Yes (1)

○ No (2)

Q22 **On what surfaces do you train/run?**

	Often (1)	Sometimes (2)	Rarely (3)
Dirt roads (trails) (1)	○	○	○
Street (tarred/paved surfaces) (2)	○	○	○
Grass (3)	○	○	○
Athletic track (tartan) (4)	○	○	○
Treadmill (5)	○	○	○

Q24 **For what time period have you been actively participating in RUNNING as a sport?**

▼ Select time (1) ... 10 years or more (12)

Q28 **For what time period have you been actively participating in TRAIL RUNNING as a sport?**

▼ Select time (1) ... 10 years or more (12)

Q26 **Did you receive any advice regarding trail running shoes before?**

- ○ Yes (1)
- ○ No (2)

Q30 If yes, what advice did you receive?

- ☐ Anti-pronation (1)
- ☐ Anti-supination (2)
- ☐ Neutral shoe (3)
- ☐ Ankle support (4)
- ☐ Orthotics (5)
- ☐ Raised heel (6)
- ☐ Front-foot support (7)
- ☐ For increased weight (8)

Q29 What brand shoe do you use most often during trail running?

▼ Adidas (1) ... Other (20)

Q29 **Did you sustain any running related injuries in the PAST TWELVE MONTHS?**

- ○ Yes (1)
- ○ No (2)

Q30 **If yes, do you still at this stage experience similar symptoms from this injury?**

- ◯ Yes (1)
- ◯ No (2)

Q31 **Please specify the DIAGNOSIS of this injury (or location of symptoms if diagnosis was not made)**

- ☐ Diagnosis (1) ____
- ☐ Location of injury (2) ____

Q32 **Are you suffering from any CHRONIC DISEASES i.e. hypertension, asthma, diabetes, cholesterol?**

- ◯ Yes (1)
- ◯ No (2)

Q33 **If yes, please list your chronic diseases.**

____

Q32 **Please list all other MEDICATIONS and/or SUPPLEMENTS that you currently use.**

____

End of Block: Demographic data
