# Supplementary material for: Epidemiology, Clinical Characteristics, and Risk Factors for Running-Related Injuries among South African Trail Runners
Source: Int J Environ Res Public Health. 2021 Nov 30;18(23):12620. doi: 10.3390/ijerph182312620 (PMC8656810; doi:10.3390/ijerph182312620)
Supplement: Supplementary file 1 [file ijerph-18-12620-s001.zip › Online supplementary File S2.pdf]

Modified OSTRC-H

Survey Flow

Block: OSTRC (1 Question)
Block: Training data (10 Questions)
Block: Injury/Illness Effect (5 Questions)

Branch: New Branch
If
If Was an INJURY responsible for your difficulty in running participation over the PAST TWO WEEKS? No Is Selected

Block: Illness ? (1 Question)

Branch: New Branch
If
If Was an ILLNESS responsible for your difficulty in running participation over the PAST TWO WEEKS? Yes Is Selected

Block: Illness 1 (9 Questions)

Branch: New Branch
If
If Do you have any OTHER ILLNESS to record? Yes Is Selected

Block: Illness 2 (9 Questions)

Branch: New Branch
If
If Do you have any OTHER ILLNESS to record? Yes Is Selected

Block: Illness 3 (8 Questions)

EndSurvey:

EndSurvey:

EndSurvey:

EndSurvey:

Block: Injury 1 (8 Questions)

Branch: New Branch
If
If Do you have any OTHER INJURY to record? Yes Is Selected

Block: Injury 2 (8 Questions)

Branch: New Branch
If
If Do you have any OTHER INJURY to record? Yes Is Selected

Block: Injury 3 (7 Questions)

Block: Illness ? (1 Question)

Branch: New Branch
If
If Was an ILLNESS responsible for your difficulty in running participation over the PAST TWO WEEKS? Yes Is Selected

Block: Illness 1 (9 Questions)

Branch: New Branch
If
If Do you have any OTHER ILLNESS to record? Yes Is Selected

Block: Illness 2 (9 Questions)

Branch: New Branch
If
If Do you have any OTHER ILLNESS to record? Yes Is Selected

Block: Illness 3 (8 Questions)

EndSurvey:

EndSurvey:

EndSurvey:

EndSurvey:

Page Break

Start of Block: OSTRC

Q1 Please answer all questions regardless of whether or not you have experienced health problems in the **PAST TWO WEEKS**.
If you have several illness or injury problems, please refer to the one that has been your worst problem in the **PAST TWO WEEKS**. You will have a chance to register other problems at the end of the questionnaireTop of Form **Let's start!**

End of Block: OSTRC

Start of Block: Training data

Q63 Initials & Surname

____

*

Q68 ID number

____

Q57 How many **running** sessions did you do in the PAST TWO WEEKS?

▼ 0 (1) ... more than 14 (16)

Q58 How many of these running sessions were **ran on trails** in the PAST TWO WEEKS?

▼ 0 (1) ... more than 14 (16)

Q59 What **distance (km)** did you run over the PAST TWO WEEKS?

____

Q82 What was the **total ascent (m)** you got during your runs over the PAST TWO WEEKS?

____

Q68 What was the total descent (m) you got during your runs over the PAST TWO WEEKS?

____

Q70 At what **average altitude (m)** did you train during the PAST TWO WEEKS?

____

Q65 What was your **average running pace (min/km**) over the PAST TWO WEEKS?

▼ 3:30 (1) ... Did not run (29)

Q60 What **cross training** did you do and **for how many hours** in the PAST TWO WEEKS?
Cycling : _______ (1)
Strength training : _______ (2)
Rowing : _______ (3)
Swimming : _______ (4)
Pilates : _______ (5)
Functional training : _______ (6)
Other sports (squash, tennis, soccer etc.) : _______ (7)
None : _______ (8)
Total : ________

End of Block: Training data

Start of Block: Injury/Illness Effect

Q2 To what extent have you **MODIFIED YOUR TRAINING OR COMPETITION** due to injury, illness or other health problems during the PAST TWO WEEKS?

- ◯ No modification (1)
- ◯ To a minor extent (2)
- ◯ To a moderate extent (3)
- ◯ To a major extend (4)
- ◯ Could not participate at all (5)

Skip To: End of Survey If To what extent have you MODIFIED YOUR TRAINING OR COMPETITION due to injury, illness or other hea... = No modification

Q3 To what extent has injury, illness or other health problems affected your **PERFORMANCE** during the PAST TWO WEEKS?

- ◯ No effect (1)
- ◯ To a minor extent (2)
- ◯ To a moderate extent (3)
- ◯ To a major extend (4)
- ◯ Could not participate at all (5)

Q4 To what extent have you **experienced symptoms/health complaints** during the PAST TWO WEEKS?

- ◯ No symptoms/health complaints (1)
- ◯ To a minor extent (2)
- ◯ To a moderate extent (3)
- ◯ To a major extend (4)
- ◯ Could not participate at all (5)

Q5 To what extent have you experienced **PAIN** related to your sport during the PAST TWO WEEKS?

- ◯ No pain (1)
- ◯ Mild pain (2)
- ◯ Moderate pain (3)
- ◯ Severe pain (4)
- ◯ Could not participate at all (5)

Q6 Was an **INJURY** responsible for your difficulty in running participation over the PAST TWO WEEKS?

- ◯ Yes (1)
- ◯ No (2)

Skip To: End of Block If Was an INJURY responsible for your difficulty in running participation over the PAST TWO WEEKS? = No

End of Block: Injury/Illness Effect

Start of Block: Illness ?

Q29 Was an **ILLNESS** responsible for your difficulty in running participation over the PAST TWO WEEKS?

○ Yes (1)

○ No (2)

Skip To: End of Survey If Was an ILLNESS responsible for your difficulty in running participation over the PAST TWO WEEKS? = No

End of Block: Illness ?

Start of Block: Illness 1

Q67 **Is this the first time you have registered this illness through this monitoring system?**

○ Yes (1)

○ No, I have reported the same problem in the previous 4 weeks (2)

○ No, I have reported the same problem previously, but it was more than 4 weeks ago (3)

Q30 **Please check the boxes corresponding to the major symptoms you have experienced during the PAST TWO WEEKS. You may select several alternatives.**

- ☐ Fever (1)
- ☐ Fatigue/malaise (2)
- ☐ Swollen glands (3)
- ☐ Sore throat (4)
- ☐ Blocked nose/running nose/sneezing (5)
- ☐ Cough (6)
- ☐ Breathing difficulty/tightness (7)
- ☐ Nausea (8)
- ☐ Vomiting (9)
- ☐ Diarrhoea (10)
- ☐ Constipation (11)
- ☐ Abdominal pain (12)
- ☐ Irregular pulse/arrhythmia (13)
- ☐ Chest pain/Angina (14)
- ☐ Other pain (15)
- ☐ Headache (16)
- ☐ Fainting (17)
- ☐ Numbness/pins and needles (18)
- ☐ Sunburn (19)

☐ Rash with itchiness (20)

☐ Ear symptoms (21)

☐ Eye symptoms (22)

☐ Symptoms from urinary tract/genitalia (23)

☐ Anxiety (24)

☐ Depression/sadness (25)

☐ Irritability (26)

☐ Muscle Cramps – Generalised (unspecific region of the body) (27)

☐ Muscle Cramps – Localised (in specific location) (28)

☐ Other (please specify) (29) ____

Q31 **Please indicate the body system involved with your illness.**

- ○ Brain and Nervous system (1)
- ○ Heart and Blood vessels (2)
- ○ Lungs and Respiratory tract (3)
- ○ Digestive system (4)
- ○ Kidney and bladder (5)
- ○ Muscle (i.e. muscle cramps, muscle weakness) (6)
- ○ Bone (i.e. osteopenia, osteoporosis, low bone density) (7)
- ○ Immune (i.e. infections) (8)
- ○ Metabolic or Endocrine (i.e. glands, hormones) (9)
- ○ Skin (10)
- ○ Do not know (11)

Q35 **Do have a specific diagnosis for your illness? Please specify**

- ○ Yes (please specify) (1) ____
- ○ No (2)

Q36 **Who made the diagnosis of your illness?**

- ○ Doctor (1)
- ○ Physiotherapist (2)
- ○ Other health care professional (3)
- ○ Coach (4)
- ○ Self-diagnosed (5)

Q37 **Did your illness have a GRADUAL or SUDDEN onset?**

- ○ Gradual (1)
- ○ Sudden (2)

Q38 **How was your illness treated or managed?**

- ☐ Self-medicated (1)
- ☐ Antibiotics (2)
- ☐ Referral to other health care professional (3)
- ☐ Other drug therapies (4)

Q40 **Please state the NUMBER OF DAYS that you had to completely miss training/races due to this illness.**

____

Q41 **Do you have any OTHER ILLNESS to record?**

- Yes (1)
- No (2)

Skip To: End of Survey If Do you have any OTHER ILLNESS to record? = No

End of Block: Illness 1

Start of Block: Illness 2

Q68 **Is this the first time you have registered this illness through this monitoring system?**

- Yes (1)
- No, I have reported the same problem in the previous 4 weeks (2)
- No, I have reported the same problem previously, but it was more than 4 weeks ago (3)

Q40 **Please check the boxes corresponding to the major symptoms you have experienced during the PAST TWO WEEKS. You may select several alternatives.**

- ☐ Fever (1)
- ☐ Fatigue/malaise (2)
- ☐ Swollen glands (3)
- ☐ Sore throat (4)
- ☐ Blocked nose/running nose/sneezing (5)
- ☐ Cough (6)
- ☐ Breathing difficulty/tightness (7)
- ☐ Nausea (8)
- ☐ Vomiting (9)
- ☐ Diarrhoea (10)
- ☐ Constipation (11)
- ☐ Abdominal pain (12)
- ☐ Irregular pulse/arrhythmia (13)
- ☐ Chest pain/Angina (14)
- ☐ Other pain (15)
- ☐ Headache (16)
- ☐ Fainting (17)
- ☐ Numbness/pins and needles (18)
- ☐ Sunburn (19)

☐ Rash with itchiness (20)

☐ Ear symptoms (21)

☐ Eye symptoms (22)

☐ Symptoms from urinary tract/genitalia (23)

☐ Anxiety (24)

☐ Depression/sadness (25)

☐ Irritability (26)

☐ Muscle Cramps – Generalised (unspecific region of the body) (27)

☐ Muscle Cramps – Localised (in specific location) (28)

☐ Other (please specify) (29) ____

Q41 **Please indicate the body system involved with your illness.**

○ Brain and Nervous system (1)

○ Heart and Blood vessels (2)

○ Lungs and Respiratory tract (3)

○ Digestive system (4)

○ Kidney and bladder (5)

○ Muscle (i.e. muscle cramps, muscle weakness) (6)

○ Bone (i.e. osteopenia, osteoporosis, low bone density) (7)

○ Immune (i.e. infections) (8)

○ Metabolic or Endocrine (i.e. glands, hormones) (9)

○ Skin (10)

○ Do not know (11)

Q42 **Do have a specific diagnosis for your illness? Please specify**

○ Yes (please specify) (1) ____

○ No (2)

Q43 **Who made the diagnosis of your illness?**

- ◯ Doctor (1)
- ◯ Physiotherapist (2)
- ◯ Other health care professional (3)
- ◯ Coach (4)
- ◯ Self-diagnosed (5)

Q44 **Did your illness have a GRADUAL or SUDDEN onset?**

- ◯ Gradual (1)
- ◯ Sudden (2)

Q45 **How was your illness treated or managed?**

- ☐ Self-medicated (1)
- ☐ Antibiotics (2)
- ☐ Referral to other health care professional (3)
- ☐ Other drug therapies (4)

Q46 **Please state the NUMBER OF DAYS that you had to completely miss training/races due to this illness.**

____

Q47 **Do you have any OTHER ILLNESS to record?**

- ○ Yes (1)
- ○ No (2)

End of Block: Illness 2

Start of Block: Illness 3

Q69 **Is this the first time you have registered this illness through this monitoring system?**

- ○ Yes (1)
- ○ No, I have reported the same problem in the previous 4 weeks (2)
- ○ No, I have reported the same problem previously, but it was more than 4 weeks ago (3)

Q48 **Please check the boxes corresponding to the major symptoms you have experienced during the PAST TWO WEEKS. You may select several alternatives.**

- ☐ Fever (1)
- ☐ Fatigue/malaise (2)
- ☐ Swollen glands (3)
- ☐ Sore throat (4)
- ☐ Blocked nose/running nose/sneezing (5)
- ☐ Cough (6)
- ☐ Breathing difficulty/tightness (7)
- ☐ Nausea (8)
- ☐ Vomiting (9)
- ☐ Diarrhoea (10)
- ☐ Constipation (11)
- ☐ Abdominal pain (12)
- ☐ Irregular pulse/arrhythmia (13)
- ☐ Chest pain/Angina (14)
- ☐ Other pain (15)
- ☐ Headache (16)
- ☐ Fainting (17)
- ☐ Numbness/pins and needles (18)
- ☐ Sunburn (19)

☐ Rash with itchiness (20)

☐ Ear symptoms (21)

☐ Eye symptoms (22)

☐ Symptoms from urinary tract/genitalia (23)

☐ Anxiety (24)

☐ Depression/sadness (25)

☐ Irritability (26)

☐ Muscle Cramps – Generalised (unspecific region of the body) (27)

☐ Muscle Cramps – Localised (in specific location) (28)

☐ Other (please specify) (29) ____

Q49 **Please indicate the body system involved with your illness.**

- ◯ Brain and Nervous system (1)
- ◯ Heart and Blood vessels (2)
- ◯ Lungs and Respiratory tract (3)
- ◯ Digestive system (4)
- ◯ Kidney and bladder (5)
- ◯ Muscle (i.e. muscle cramps, muscle weakness) (6)
- ◯ Bone (i.e. osteopenia, osteoporosis, low bone density) (7)
- ◯ Immune (i.e. infections) (8)
- ◯ Metabolic or Endocrine (i.e. glands, hormones) (9)
- ◯ Skin (10)
- ◯ Do not know (11)

Q50 **Do have a specific diagnosis for your illness? Please specify**

- ◯ Yes (please specify) (1) ____
- ◯ No (2)

Q51 **Who made the diagnosis of your illness?**

- ○ Doctor (1)
- ○ Physiotherapist (2)
- ○ Other health care professional (3)
- ○ Coach (4)
- ○ Self-diagnosed (5)

Q52 **Did your illness have a GRADUAL or SUDDEN onset?**

- ○ Gradual (1)
- ○ Sudden (2)

Q53 **How was your illness treated or managed?**

- ☐ Self-medicated (1)
- ☐ Antibiotics (2)
- ☐ Referral to other health care professional (3)
- ☐ Other drug therapies (4)

Q54 **Please state the NUMBER OF DAYS that you had to completely miss training/races due to this illness.**

____

End of Block: Illness 3

Start of Block: Injury 1

Q64 Is this the **first time** you have **registered this injury** through this monitoring system?

- Yes (1)
- No, I have reported the same problem in the previous 4 weeks (2)
- No, I have reported the same problem previously, but it was more than 4 weeks ago (3)

Q7 Please select the box that best describes the **LOCATION** of your injury. If the injury involves several locations please select the main area.
If you have **multiple injuries** please complete a **separate registration** of each one.

- ◯ Head/face (1)
- ◯ Neck (2)
- ◯ Shoulder (including clavicle) (3)
- ◯ Upper arm (4)
- ◯ Elbow (5)
- ◯ Forearm (6)
- ◯ Wrist (7)
- ◯ Hand/fingers (8)
- ◯ Chest/ribs (9)
- ◯ Abdomen (10)
- ◯ Thoracic spine (11)
- ◯ Lumbar spine (12)
- ◯ Pelvis/buttock (13)
- ◯ Hip/groin (14)
- ◯ Thigh (front - quadricep) (15)
- ◯ Thigh (back - hamstring) (16)
- ◯ Knee (17)
- ◯ Lower leg (18)
- ◯ Ankle (19)
- ◯ Foot/toes (20)

Q8 Please select a box that best describes your **TYPE OF INJURY.**

○ Concussion (symptoms like disorientation, dizziness, loss of memory, nausea or vomiting due to a blow to the head) (1)

○ Fracture (traumatic - broken bone caused by sudden impact) (2)

○ Stress fracture (overuse - fracture in a weight bearing bone caused by repetitive stress (e.g. running), a stress fracture in one of the small bones in the foot will typically cause severe pain at the beginning of a run, moderate pain during the run and severe pain at the end and after the run) (3)

○ Other bone injuries (4)

○ Dislocation, subluxation (the total or partial displacement or misalignment of bones in a joint, most often caused by a sudden impact to the joint) (5)

○ Tendon rupture (tearing of a tendon that occurs when the forces placed upon the tendon exceed its tensile strength) (6)

○ Tendinosis/tendinopathy (all non-inflammatory and inflammatory conditions affecting a tendon, "tendinitis") (7)

○ Ligamentous rupture (tearing of the bands of fibrous tissue connecting bones or cartilages, serving to support and strengthen joints) (8)

○ Sprain (wrenching or twisting of a joint, with partial rupture of its ligaments, accompanied by severe pain, impaired function, swelling, heat and discolouration of the skin) (9)

○ Lesion of meniscus or cartilage (injuries of meniscus [knee] or joint surfaces) (10)

○ Muscle strain (11)

○ Muscle rupture/tear (12)

○ Contusion/haematoma/bruise (13)

○ Arthritis/synovitis/bursitis (inflammation of any part of a joint or structures near the joint, characterize by pain on movement, tenderness, heat and swelling) (14)

○ Fasciitis/aponeurosis injury (inflammation or injury of a sheet like tendinous expansion, e.g. plantar fasciitis) (15)

○ Impingement (compression of a nerve, blood vessel, tendon, ligament or muscle through a constricted space, e.g. sciatica) (16)

- Skin laceration/cut/lesion (17)
- Skin abrasion/chafing (18)
- Dental injury/broken tooth (19)
- Nerve injury/spinal cord injury (20)
- Muscle cramps or spasm (21)
- I don't know (22)
- Other (please specify) (23) ____

Q9 **Who** made the **diagnosis** of your injury?

- Doctor (1)
- Physiotherapist (2)
- Other health care professional (3)
- Coach (4)
- Self-diagnosed (5)

Q10 Did your injury have a **GRADUAL** or **SUDDEN** onset?

- Gradual (1)
- Sudden (2)

Q11 Was the **injury due to a specific action?** (fall, jump, landing, increased pace, overstretch, collision etc.)

◯ Yes (please specify the action) (1) ____

◯ No (2)

Q14 Please state the **NUMBER OF DAYS** that you had to completely **miss training/races** due to this injury.

____

Q15 Do you have any **OTHER INJURY** to record?

◯ Yes (1)

◯ No (2)

End of Block: Injury 1

Start of Block: Injury 2

Q83 Is this the **first time** you have **registered this injury** through this monitoring system?

◯ Yes (1)

◯ No, I have reported the same problem in the previous 4 weeks (2)

◯ No, I have reported the same problem previously, but it was more than 4 weeks ago (3)

Q84 Please select the box that best describes the **LOCATION** of your injury. If the injury involves several locations please select the main area.
If you have **multiple injuries** please complete a **separate registration** of each one.

- ◯ Head/face (1)
- ◯ Neck (2)
- ◯ Shoulder (including clavicle) (3)
- ◯ Upper arm (4)
- ◯ Elbow (5)
- ◯ Forearm (6)
- ◯ Wrist (7)
- ◯ Hand/fingers (8)
- ◯ Chest/ribs (9)
- ◯ Abdomen (10)
- ◯ Thoracic spine (11)
- ◯ Lumbar spine (12)
- ◯ Pelvis/buttock (13)
- ◯ Hip/groin (14)
- ◯ Thigh (front - quadricep) (15)
- ◯ Thigh (back - hamstring) (16)
- ◯ Knee (17)
- ◯ Lower leg (18)
- ◯ Ankle (19)
- ◯ Foot/toes (20)

Q85 Please select a box that best describes your **TYPE OF INJURY.**

- ◯ Concussion (symptoms like disorientation, dizziness, loss of memory, nausea or vomiting due to a blow to the head) (1)
- ◯ Fracture (traumatic - broken bone caused by sudden impact) (2)
- ◯ Stress fracture (overuse - fracture in a weight bearing bone caused by repetitive stress (e.g. running), a stress fracture in one of the small bones in the foot will typically cause severe pain at the beginning of a run, moderate pain during the run and severe pain at the end and after the run) (3)
- ◯ Other bone injuries (4)
- ◯ Dislocation, subluxation (the total or partial displacement or misalignment of bones in a joint, most often caused by a sudden impact to the joint) (5)
- ◯ Tendon rupture (tearing of a tendon that occurs when the forces placed upon the tendon exceed its tensile strength) (6)
- ◯ Tendinosis/tendinopathy (all non-inflammatory and inflammatory conditions affecting a tendon, "tendinitis") (7)
- ◯ Ligamentous rupture (tearing of the bands of fibrous tissue connecting bones or cartilages, serving to support and strengthen joints) (8)
- ◯ Sprain (wrenching or twisting of a joint, with partial rupture of its ligaments, accompanied by severe pain, impaired function, swelling, heat and discolouration of the skin) (9)
- ◯ Lesion of meniscus or cartilage (injuries of meniscus [knee] or joint surfaces) (10)
- ◯ Muscle strain (11)
- ◯ Muscle rupture/tear (12)
- ◯ Contusion/haematoma/bruise (13)
- ◯ Arthritis/synovitis/bursitis (inflammation of any part of a joint or structures near the joint, characterize by pain on movement, tenderness, heat and swelling) (14)
- ◯ Fasciitis/aponeurosis injury (inflammation or injury of a sheet like tendinous expansion, e.g. plantar fasciitis) (15)
- ◯ Impingement (compression of a nerve, blood vessel, tendon, ligament or muscle through a constricted space, e.g. sciatica) (16)

○ Skin laceration/cut/lesion (17)

○ Skin abrasion/chafing (18)

○ Dental injury/broken tooth (19)

○ Nerve injury/spinal cord injury (20)

○ Muscle cramps or spasm (21)

○ I don't know (22)

○ Other (please specify) (23) ____

Q86 **Who** made the **diagnosis** of your injury?

○ Doctor (1)

○ Physiotherapist (2)

○ Other health care professional (3)

○ Coach (4)

○ Self-diagnosed (5)

Q87 Did your injury have a **GRADUAL** or **SUDDEN** onset?

○ Gradual (1)

○ Sudden (2)

Q88 Was the **injury due to a specific action?** (fall, jump, landing, increased pace, overstretch, collision etc.)

○ Yes (please specify the action) (1) ____

○ No (2)

Q89 Please state the **NUMBER OF DAYS** that you had to completely **miss training/races** due to this injury.

____

Q90 Do you have any **OTHER INJURY** to record?

○ Yes (1)

○ No (2)

End of Block: Injury 2

Start of Block: Injury 3

Q99 Is this the **first time** you have **registered this injury** through this monitoring system?

○ Yes (1)

○ No, I have reported the same problem in the previous 4 weeks (2)

○ No, I have reported the same problem previously, but it was more than 4 weeks ago (3)

Q100 Please select the box that best describes the **LOCATION** of your injury. If the injury involves several locations please select the main area.
If you have **multiple injuries** please complete a **separate registration** of each one.

- ◯ Head/face (1)
- ◯ Neck (2)
- ◯ Shoulder (including clavicle) (3)
- ◯ Upper arm (4)
- ◯ Elbow (5)
- ◯ Forearm (6)
- ◯ Wrist (7)
- ◯ Hand/fingers (8)
- ◯ Chest/ribs (9)
- ◯ Abdomen (10)
- ◯ Thoracic spine (11)
- ◯ Lumbar spine (12)
- ◯ Pelvis/buttock (13)
- ◯ Hip/groin (14)
- ◯ Thigh (front - quadricep) (15)
- ◯ Thigh (back - hamstring) (16)
- ◯ Knee (17)
- ◯ Lower leg (18)
- ◯ Ankle (19)
- ◯ Foot/toes (20)

Q101 Please select a box that best describes your **TYPE OF INJURY.**

○ Concussion (symptoms like disorientation, dizziness, loss of memory, nausea or vomiting due to a blow to the head) (1)

○ Fracture (traumatic - broken bone caused by sudden impact) (2)

○ Stress fracture (overuse - fracture in a weight bearing bone caused by repetitive stress (e.g. running), a stress fracture in one of the small bones in the foot will typically cause severe pain at the beginning of a run, moderate pain during the run and severe pain at the end and after the run) (3)

○ Other bone injuries (4)

○ Dislocation, subluxation (the total or partial displacement or misalignment of bones in a joint, most often caused by a sudden impact to the joint) (5)

○ Tendon rupture (tearing of a tendon that occurs when the forces placed upon the tendon exceed its tensile strength) (6)

○ Tendinosis/tendinopathy (all non-inflammatory and inflammatory conditions affecting a tendon, "tendinitis") (7)

○ Ligamentous rupture (tearing of the bands of fibrous tissue connecting bones or cartilages, serving to support and strengthen joints) (8)

○ Sprain (wrenching or twisting of a joint, with partial rupture of its ligaments, accompanied by severe pain, impaired function, swelling, heat and discolouration of the skin) (9)

○ Lesion of meniscus or cartilage (injuries of meniscus [knee] or joint surfaces) (10)

○ Muscle strain (11)

○ Muscle rupture/tear (12)

○ Contusion/haematoma/bruise (13)

○ Arthritis/synovitis/bursitis (inflammation of any part of a joint or structures near the joint, characterize by pain on movement, tenderness, heat and swelling) (14)

○ Fasciitis/aponeurosis injury (inflammation or injury of a sheet like tendinous expansion, e.g. plantar fasciitis) (15)

○ Impingement (compression of a nerve, blood vessel, tendon, ligament or muscle through a constricted space, e.g. sciatica) (16)

Skin laceration/cut/lesion (17)

Skin abrasion/chafing (18)

Dental injury/broken tooth (19)

Nerve injury/spinal cord injury (20)

Muscle cramps or spasm (21)

I don't know (22)

Other (please specify) (23) ____

Q102 **Who** made the **diagnosis** of your injury?

Doctor (1)

Physiotherapist (2)

Other health care professional (3)

Coach (4)

Self-diagnosed (5)

Q103 Did your injury have a **GRADUAL** or **SUDDEN** onset?

Gradual (1)

Sudden (2)

Q104 Was the **injury due to a specific action?** (fall, jump, landing, increased pace, overstretch, collision etc.)

○ Yes (please specify the action) (1) ____

○ No (2)

Q105 Please state the **NUMBER OF DAYS** that you had to completely **miss training/races** due to this injury.

____

End of Block: Injury 3
