# Supplementary material for: Epidemiology, Clinical Characteristics, and Risk Factors for Running-Related Injuries among South African Trail Runners
Source: Int J Environ Res Public Health. 2021 Nov 30;18(23):12620. doi: 10.3390/ijerph182312620 (PMC8656810; doi:10.3390/ijerph182312620)
Supplement: Supplementary file 1 [file ijerph-18-12620-s001.zip › Online supplementary File S3.pdf]

ONLINE SUPPLEMENTARY MATERIAL (S3)

Epidemiology, clinical characteristics, and risk factors for running-related injuries among trail runners

Table S1: The frequency of tissue and pathology types of RRIs among trail runners (% of RRIs) (*n*=205)

Tissue	Pathology type	*n*	% of all injuries (*n*=205)
Muscle/Tendon	**All**	**108**	**52.7**
	Muscle injury	42	20.5
	Muscle cramps	6	2.9
	Tendinopathy	57	27.8
	Tendon rupture	3	1.5
Nervous	**All**	**2**	**1.0**
	Peripheral nerve injury	2	1.0
Bone	**All**	**10**	**4.9**
	Bone stress injury	6	2.9
	Bone contusion	1	0.5
	Other bone injuries	3	1.5
Cartilage/Synovium/Bursa	**All**	**16**	**7.8**
	Cartilage injury	5	2.4
	Synovitis/Capsulitis/Bursitis	11	5.4
Ligament/Joint capsule	**All**	**18**	**8.8**
	Joint sprain (ligament tear or acute instability episode)	18	8.8
Superficial tissues/skin	**All**	**10**	**4.9**
	Contusion (superficial)	5	2.4
	Laceration	3	1.5
	Abrasion	1	0.5
	Blisters	1	0.5
Other		**20**	**9.8**
Missing		**21**	**10.2**
